# Supplementary material for: Identification of photoperiod-induced specific miRNAs in the adrenal glands of Sunite sheep (Ovis aries)
Source: Front Vet Sci. 2022 Jul 22;9:888207. doi: 10.3389/fvets.2022.888207 (PMC9354845; doi:10.3389/fvets.2022.888207)
Supplement: Supplementary file 1 [file Data_Sheet_1.ZIP › Supplementary file/miRDeep2 file.zip!/miRDeep2 file/Know_Structure/LP42A1/oar-mir-10b.pdf]

miRBase precursor : oar-mir-10b
Total read count : 880688
oar-miR-10b read count : 877900
remaining reads : 2788

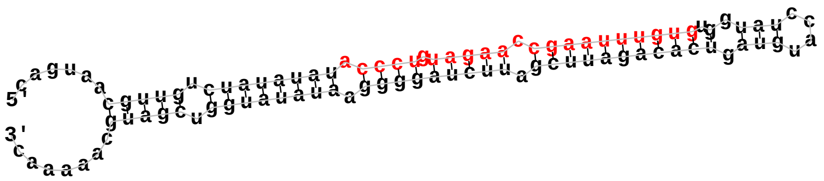

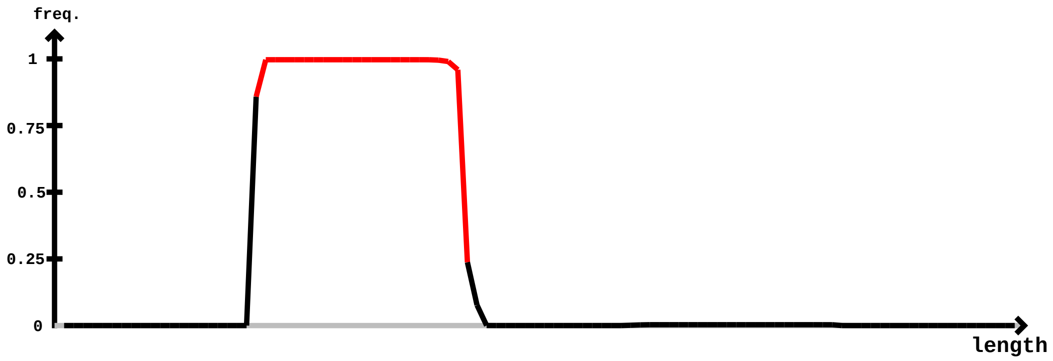

oar-miR-10b

```
5'- caguaacguugucuauauauacccuguagaaccgaauuugugugguauccauguagucacagauucgauucuaggggaauauauggucgaugcaaaaac   -3'    exp
    ......(((((.((((((((.((((.(((((.((((((((((.(.(((....))).))))))))))).))))))))).)))))))).))))).......     reads      mm        sample
    .................uauacccuguagaaccgaauuugu..........................................................     2          0         seq
    ..................auacccuguagaaccgaauuug...........................................................     1          0         seq
    ..................auacccuguagaaccgaauuugu..........................................................     12         0         seq
    ..................auacccuguagaaccgaauuugug.........................................................     1          0         seq
    ..................auacccuguagaaccgaauuugugu........................................................     1          0         seq
    ...................uacccuguagaaccgaa...............................................................     15         0         seq
    ...................uacccuguagaaccgaau..............................................................     99         0         seq
    ...................uacccuguagaaccgaauu.............................................................     1188       0         seq
    ...................uacccuguagaaccgaauuu............................................................     4059       0         seq
    ...................uacccuguagaaccgaauuug...........................................................     24949      0         seq
    ...................uacccuguagaaccgaauuugu..........................................................     595040     0         seq
    ...................uacccuguagaaccgaauuugug.........................................................     102379     0         seq
    ...................uacccuguagaaccgaauuugugu........................................................     28224      0         seq
    ...................uacccuguagaaccgaauuugugug.......................................................     27         0         seq
    ...................uacccuguagaaccgaauuugugugg......................................................     4          0         seq
    ...................uacccuguagaaccgaauuugugugguauc..................................................     1          0         seq
    ....................acccuguagaaccgaau..............................................................     4          0         seq
    ....................acccuguagaaccgaauu.............................................................     107        0         seq
    ....................acccuguagaaccgaCuuu............................................................     1          1         seq
    ....................acccuguagaaccgaauuu............................................................     482        0         seq
    ....................acccuguagaaccgaauuug...........................................................     2249       0         seq
    ....................acccuguagaaccgaauuugu..........................................................     40635      0         seq
    ....................acccuguagaaccgaauuugug.........................................................     39092      0         seq
    ....................acccuguagaaccgaauuugugu........................................................     39091      0         seq
    ....................acccuguagaaccgaauuugugug.......................................................     136        0         seq
    ....................acccuguagaaccgaauuugugugg......................................................     10         0         seq
    ....................acccuguagaaccgaauuuguguggu.....................................................     1          0         seq
    .....................cccuguagaaccgaauuug...........................................................     1          0         seq
    .....................cccuguagaaccgaauuugu..........................................................     14         0         seq
    .....................cccuguagaaccgaauuugug.........................................................     11         0         seq
    .....................cccuguagaaccgaauuugugu........................................................     40         0         seq
    ......................ccuguagaaccgaauuug...........................................................     1          0         seq
    ......................ccuguagaaccgaauuugu..........................................................     11         0         seq
    ......................ccuguagaaccgaauuugug.........................................................     7          0         seq
    ......................ccuguagaaccgaauuugugu........................................................     4          0         seq
```

oar-miR-10b

```
caguaacguugucuauauauacccuguagaaccgaauuugugugguauccauguagucacagauucgauucuaggggaauauauggucgaugcaaaaac
.......................cuguagaaccgaauuugug.........................................................	1	0	seq
.......................cuguagaaccgaauuugugu........................................................	3	0	seq
........................................................ucacagauucgauucuagggga.....................	1	0	seq
........................................................ucacagauucgauucuaggggaa....................	1	0	seq
.........................................................cacagauucgauucuagggga.....................	1	0	seq
.........................................................cacagauucgauucuaggggaa....................	3	0	seq
.........................................................cacagauucgauucuaggggaau...................	11	0	seq
..........................................................acagauucgauucuagggg......................	3	0	seq
..........................................................acagauucgauucuagggga.....................	40	0	seq
..........................................................acagauucgauucuaggggaa....................	44	0	seq
..........................................................acagauucgauucuaggggaau...................	977	0	seq
...........................................................cagauucgauucuagggg......................	2	0	seq
...........................................................cagauucgauucuagggga.....................	28	0	seq
...........................................................cagauucgauucuaggggaa....................	30	0	seq
...........................................................cagauucgauucuaggggaau...................	1071	0	seq
...........................................................cagauucgauucuaggggaaua..................	9	0	seq
...........................................................cagauucgauucuaggggaauau.................	4	0	seq
............................................................agauucgauucuagggg......................	1	0	seq
............................................................agauucgauucuagggga.....................	6	0	seq
............................................................agauucgauucuaggggaa....................	7	0	seq
............................................................agauucgauucuaggggaau...................	490	0	seq
............................................................agauucgauucuaggggaaua..................	24	0	seq
............................................................agauucgauucuaggggaauau.................	30	0	seq
............................................................agauucgauucuaggggaauaua................	1	0	seq
.............................................................gauucgauucuaggggaau...................	1	0	seq
```
